# A multi-centre observational cohort study on pharmacogenomic predictors of rosuvastatin discontinuation in a multiethnic population

**DOI:** 10.3389/fphar.2025.1656520

**Published:** 2025-08-29

**Authors:** Mais N. Alqasrawi, Zeina N. Al-Mahayri, Lubna Q. Khasawneh, Areej S. AlBawa’neh, Lilas Dabaghie, Sahar M. Altoum, Dana Hamza, Salahdein Aburuz, Virendra Misra, Gohar Jamil, Husam Ouda, Faiz Al-Bakshy, Khuzama AlAhamad, Mohammad M. Al-Ahmad, Fatima Al-Maskari, Juma AlKaabi, George P. Patrinos, Bassam R. Ali

**Affiliations:** ^1^ Department of Genetics and Genomics, College of Medicine and Health Sciences, United Arab Emirates University, Al-Ain, United Arab Emirates; ^2^ Department of Biomedical Sciences, College of Health Sciences, Abu Dhabi University, Abu Dhabi, United Arab Emirates; ^3^ Department of Pharmacology and Therapeutics, College of Medicine and Health Science, United Arab Emirates University, Al-Ain, United Arab Emirates; ^4^ Burjeel Day Surgery Centre, Abu Dhabi, United Arab Emirates; ^5^ Department of Cardiology, Tawam Hospital, Al-Ain, United Arab Emirates; ^6^ The Heart Medical Centre, Al-Ain, United Arab Emirates; ^7^ Department of Cardiology, Mediclinic Al-Ain Hospital, Al-Ain, United Arab Emirates; ^8^ Department of Clinical Pharmacy, Mediclinic Al-Ain Hospital, Al-Ain, United Arab Emirates; ^9^ Department of Clinical Pharmacy, College of Pharmacy, Al-Ain University, Al-Ain, United Arab Emirates; ^10^ Zayed Centre for Health Sciences, United Arab Emirates University, Al-Ain, United Arab Emirates; ^11^ Public Health Institute, College of Medicine and Health Sciences, United Arab Emirates University, Al-Ain, United Arab Emirates; ^12^ Department of Internal Medicine, College of Medicine and Health Sciences, United Arab Emirates University, Al-Ain, United Arab Emirates; ^13^ Laboratory of Pharmacogenomics and Individualized Therapy, Department of Pharmacy, School of Health Sciences, University of Patras, Patras, Greece

**Keywords:** rosuvastatin, pharmacogenomics, ABCG2 rs2231142, personalized medicine, statin discontinuation

## Abstract

**Background:**

Rosuvastatin is widely used for cardiovascular risk reduction, but treatment discontinuation limits its long-term benefit. Genetic variants, particularly in *ABCG2* and *SLCO1B1*, influence rosuvastatin’s transport, efficacy, and tolerability. The *ABCG2* rs2231142 variant is associated with enhanced efficacy due to increased systemic exposure; however, it also raises the risk of adverse effects, especially muscle-related symptoms. Evaluating the impact of these variants in a real-world, multiethnic population is essential to improving adherence and guiding personalized therapy. The aim of this study is to investigate the influence of *ABCG2* rs2231142 (G>T; Q141K) and *SLCO1B1* rs4149056 (T>C; V174A) variants on rosuvastatin discontinuation and LDL cholesterol changes in a multiethnic population in the United Arab Emirates (UAE).

**Methods:**

In this multicenter prospective cohort study, 422 adults prescribed rosuvastatin were followed for 12 months. Discontinuation data were collected from records or phone calls. Genotyping was performed using TaqMan SNP assays. Cox regression and Kaplan-Meier analyses assessed discontinuation risk by genotype; LDL changes were analyzed using descriptive statistics and logistic regression.

**Results:**

The *ABCG2* rs2231142 T/T genotype had the highest risk of discontinuation (HR = 4.40, p < 0.001), followed by G/T (HR = 1.75). LDL change differed significantly between continuers (−17.86%) and discontinuers (+21.89%) (p < 0.001). The *ABCG2* variant was more frequent among discontinuers (30.6% vs. 17.4%, p = 0.0026). *SLCO1B1* rs4149056 was not associated with discontinuation.

**Conclusion:**

Minor allele carriers are at higher risk of discontinuation due to adverse effects. Genetic testing for *ABCG2* may support personalized rosuvastatin therapy and improve adherence.

## 1 Introduction

Statins, inhibitors of 3-hydroxy-3-methylglutaryl coenzyme A (HMG-CoA) reductase, are among the most commonly prescribed medications globally for preventing cardiovascular diseases (CVDs) (Grundy et al., 2019). Both primary and secondary prevention strategies consistently demonstrate statin efficacy in reducing cardiovascular events (Glynn, 2010). The 2018 American College of Cardiology (ACC) and American Heart Association (AHA) guidelines recommend moderate to high-intensity statin therapy for patients at increased risk of atherosclerotic cardiovascular disease. For individuals with established CVD, a 1 mmol/L reduction in low-density lipoprotein (LDL) cholesterol corresponds to a 20% reduction in the annual incidence of major vascular events (Grundy et al., 2019). Despite this robust evidence, adherence to statin therapy remains suboptimal for most populations^3,43^, often limited by adverse effects that lead to premature treatment discontinuation (Naderi et al., 2012). CVD remains the leading cause of morbidity and mortality globally including within the Middle East, with the region projected to experience one of the most dramatic global increases in atherosclerosis risk estimator (ASCVD) burden over the coming decade (Gulf Consensus, 2021). The 2022 Saudi Guidelines for Dyslipidaemia emphasize the early onset and clustering of risk factors in the region’s population, advocating more intensive lipid-lowering strategies tailored to regional risk patterns (AlRahimi et al., 2023). However, real-world studies show poor adherence to statin therapy. A recent scoping review further highlights a lack of high-quality adherence data across the Gulf Cooperation Council (GCC), underlining the need for region-specific research and interventions (Amoodi et al., 2024). Building on the 2022 Clinical Pharmacogenomics Implementation Consortium (CPIC) guidelines, which recommend *SLCO1B1* and *ABCG2* variants as PGx biomarkers to guide statin therapy and considering that *ABCG2* genetic variants primarily influence rosuvastatin (Cooper-DeHoff et al., 2022). Statins are generally well tolerated, but statin-associated musculoskeletal symptoms (SAMS) are among the most frequent causes of therapy interruption (Stroes et al., 2015). While severe adverse effects, such as rhabdomyolysis, are exceedingly rare (Graham et al., 2004), an increasing number of studies have highlighted the potential clinical impact of SAMS (Davis and Weller, 2021). While observational studies and registries report a SAMS incidence ranging from 17% to 30%, randomized controlled trials (RCTs) indicate a significantly lower rate of 4.9% (Peto and Collins, 2018; Bytyçi et al., 2022). It is essential to recognize that individuals participating in RCTs are often highly motivated and may tend to minimize or not fully report symptoms (Ward et al., 2019). These symptoms, even in the absence of elevated creatine kinase (CK) levels, are significant contributors to patient nonadherence (Reith et al., 2022). The pharmacokinetics of statins particularly systemic exposure play a pivotal role in determining their tolerability. Drug-Drug Interactions (DDIs) and Drug-Gene Interactions (DGIs) further exacerbate the risk of SAMS, highlighting the complex interplay between genetic predispositions and clinical outcomes (Birmingham et al., 2015; Arrigoni et al., 2017).

The *ABCG2 (rs2231142)* variant is a key PGx determinant of rosuvastatin exposure, impairing the function of the breast cancer resistance protein (BCRP) efflux transporter and leading to elevated plasma concentrations (Liu et al., 2024). This variant has been implicated in increased risks of adverse effects and muscle-related symptoms, both of which are frequent causes of statin discontinuation (Lehtisalo et al., 2023). Although guidelines such as those from the CPIC recommend dose adjustments for *ABCG2* variant carriers, evidence on the impact of this variant on rosuvastatin discontinuation remains limited, especially in multiethnic populations, where allele frequencies vary substantially (Cooper-DeHoff et al., 2022).

Our previous study examined these variants within the Emirati population. The findings highlighted distinct allele frequencies that significantly influence statin safety, underscoring the importance of population-specific PGx data to optimize clinical decision-making and reduce the risk of adverse drug reactions, particularly in understudied populations (Alqasrawi et al., 2024). Other studies from the UAE have similarly reported significant pharmacogenomic variability across key drug-metabolizing genes, including those involved in clopidogrel and other cardiovascular therapies (Al-Mahayri et al., 2020; Khasawneh et al., 2024).

This study aims to evaluate the association between the *ABCG2 rs2231142 (G>T; Q141K)* and *SLCO1B1 rs4149056 (T>C; V174A)* variants and rosuvastatin discontinuation in a diverse population in the UAE, leveraging a practice-based cohort with detailed genotypic and clinical data. By examining the influence of this PGx variant on adverse effects and treatment persistence, this work seeks to advance the understanding of personalized statin therapy, particularly in populations with high genetic diversity.

## 2 Materials and methods

### 2.1 Subjects and settings

This multicentre observational study was conducted in Al Ain, United Arab Emirates, as part of the *EmHeart* multicenter interventional cohort study described elsewhere (Al-Mahayri et al., 2022). Data were collected from a multi-center pharmacogenetic study conducted across healthcare institutions in the UAE between January 2021 and June 2023. Participants were recruited from multiple healthcare facilities, including Tawam Hospital (a government tertiary hospital-Cardiology unit), Mediclinic Hospital (a private multispecialty hospital-Cardiology Unit), The Heart Medical Center in Al-Ain (a private cardiovascular specialty center), and Burjeel Hospital in Abu Dhabi (a private tertiary hospital).

A total of 500 eligible participants were identified, of whom 78 were excluded due to loss to follow-up or finish the follow-up period. These individuals showed similar baseline characteristics in terms of age, sex, and genotype distribution compared to the participants who completed the study. The reasons for loss to follow-up were categorized as follows: forty participants either formally withdrew from the study or became unreachable despite repeated contact attempts; twenty participants did not attend follow-up appointments with their physicians, and no updated information could be retrieved from their medical records; and eighteen participants did not initiate rosuvastatin therapy or discontinued it within the first 7 days of use, making it inappropriate to classify them as either continuers or discontinuers. These scenarios are commonly encountered in real-world clinical research and reflect typical challenges such as patient withdrawal, disengagement, or incomplete treatment initiation.

The final analysis included 422 participants who met all eligibility criteria. Participants were followed for 12 months, during which data on treatment discontinuation were collected from Electronic Medical Records (EMRs) and via follow-up Phone calls. Discontinuation was defined as the cessation of rosuvastatin therapy for more than 30 days without switching to an alternative lipid-lowering medication, while persistence was defined as the duration of uninterrupted rosuvastatin use, measured in days. Follow-up phone calls were conducted 1, 3, 6 and 12 months after therapy initiation. During these calls, trained healthcare professionals used a standardized script to gather information on reasons for discontinuation, experienced side effects, and any alternative treatments initiated. Discontinuation rates were calculated as the proportion of patients who stopped rosuvastatin during the study period based on self-reports from follow-up calls cross-verified with pharmacy refill records.

The study population included both statin initiators and pre-existing users, who were followed for 12 months after recruitment. Statin initiators, comprising 72% of the population, were prescribed statins at recruitment and provided data on early adverse effects, such as SAMS, and discontinuation rates. Pre-existing users, already on statins, were evaluated for ongoing continuation, therapy changes, and cumulative adverse effects. This approach allowed for a comprehensive understanding of discontinuation patterns across different stages of therapy.

### 2.2 Data collection, ethical approvals, and follow-up

The study adhered to the Declaration of Helsinki and received approval from the Abu Dhabi Health Research and Technology Ethical Committee under reference numbers (DOH/CVDC/2020/1187), (DOH/CVDC/2021/1519), (DOH/CVDC/2022/1458), (MCME.CR.213.MAIN.2021), (DOH/CVDC/2023/1952), and (SNA/FA/2020-14). These approvals were granted under the *EmHeart* pharmacogenomic study framework and were used to support multiple independently designed research studies. One of these, which focused on adverse effects associated with statin use, addressed a different research objective and analytical approach (23).Data were systematically recorded using Castor-EDC software (Netherlands) (www.castoredc.com). Baseline characteristics, including age, sex, and comorbidities, were extracted from electronic medical records. Clinical information, including the rationale for statin prescriptions (e.g., CVDs) and comorbidities influencing SAMS risk (e.g., thyroid disorders), was obtained from EMRs using the International Classification of Diseases. Discontinuation data, including the date and reason, were also extracted from EMRs, with any gaps supplemented by patient follow-up calls. We further utilized Follow-ups, combined medical record reviews, and patient calls to verify SAMS occurrence within 1 year of recruitment. Concomitant medications, confirmed diagnoses, and all necessary data on demographics, laboratory results, and documented drug allergies were also collected. Selection bias was minimized by including only participants with complete genetic and clinical data. Recall and misclassification bias was addressed by validating statin discontinuation data through EMRs and follow-up calls, ensuring accurate classification. Reasons for discontinuation were collected through structured follow-up interviews and/or extracted from electronic medical records. Documented reasons included statin-associated muscle symptoms (SAMS), financial barriers, perceived therapeutic sufficiency, concerns about long-term safety, and a preference for lifestyle modification over pharmacological therapy. However, as this information was not consistently available for all discontinuing participants, we did not perform further subgroup analyses based on these reasons.

### 2.3 LDL measurements

The LDL cholesterol levels were obtained at baseline and after 1 year of follow-up. A single value was used for baseline LDL levels for all patients. At follow-up, the mean of two or three LDL measurements was calculated for all participants, ensuring a more representative measure This approach accounted for potential variability in individual measurements and provided a consistent basis for analysis. The LDL-C analysis was conducted on 100 participants with available follow-up data as part of the efficacy analysis, which requires a smaller subset of the total cohort. Due to inconsistencies in the timing of lipid testing in routine UAE healthcare settings, only 100 participants had complete LDL-C measurements at both baseline and follow-up. This subgroup was selected based solely on data availability, not pre-defined criteria. The LDL measurements were categorized based on the percentage change from baseline to follow-up, differentiating between continuers and discontinuers. Descriptive statistics were computed for the percent change in LDL levels, including means, standard deviations, and ranges LDL-C levels were measured using standardized enzymatic assays to ensure consistency across different sites.

### 2.4 Genotyping

All participants were genotyped for the *ABCG2* rs2231142 (G>T; Q141K) and *SLCO1B1* rs4149056 (T>C; V174A) variants. Peripheral blood samples were collected in EDTA-containing vacutainer tubes and stored at −20 °C until analysis. Genomic DNA was extracted using either the FlexiGene DNA kit or QIAamp DNA kit (Qiagen, Germany). Genotyping was performed using TaqMan SNP assays on the QuantStudio 7 Flex PCR system (Applied Biosystems, Thermo Fisher Scientific). Genotype calls were validated using TaqMan Genotyper software and confirmed by Sanger sequencing for a representative subset, yielding 100% concordance. In this study, the *ABCG2* variant rs2231142 is reported as G>T (Q141K), following the top (plus) strand notation used by pharmacogenomic genotyping platforms and widely adopted in the literature. Although the nucleotide change is technically c.421C>A on the coding (cDNA) strand, the G>T representation reflects the forward genomic strand and aligns with standard reporting in databases such as PharmGKB and CPIC. This notation facilitates consistency and comparability with previous pharmacogenomic studies.

### 2.5 Statistical analysis

Treatment discontinuation was analyzed using Kaplan-Meier plots and Cox proportional hazards models to compare discontinuation-free survival among participants with G/G, G/T, and T/T genotypes of the *ABCG2* variant. Discontinuation-free survival refers to the percentage of participants who remained on rosuvastatin therapy without discontinuing over the 12-month follow-up period. This measure reflects the proportion of patients persisting with treatment at various time points. Participants who did not discontinue therapy during the study were censored at the time of their last follow-up visit. Kaplan-Meier plots assessed discontinuation-free survival probabilities, while Cox proportional hazards models evaluated the risk of discontinuation across genotypic groups. Hazard ratios (HRs) and 95% confidence intervals (CIs) quantified the relative risk. Descriptive statistics were calculated for continuers and discontinuers, including mean percent change in LDL, standard deviations, and ranges. Additional comparisons were made for LDL changes by statin intensity and genotype groups using t-tests and ANOVA where appropriate. LDL analysis highlights the differences in LDL percent change between continuers and discontinuers. The assumption of normality for LDL percent change was assessed and met, justifying the use of the t-test. The analysis provided means, standard deviations, ranges, and P-values to determine the statistical significance of differences between the groups. Univariate and multivariate analyses were conducted using SPSS (version 29.0, IBM Corporation, Armonk, NY, United States), adjusting for covariates such as age, sex, and statin intensity. Statistical significance was set at p < 0.05.

## 3 Results

### 3.1 Participant characteristics

Out of the 500 enrolled participants, 422 completed the follow-up and were included in the final analysis. The remaining 78 (15.6%) were excluded due to loss to follow-up. Their baseline demographics were comparable to those of the retained cohort. Baseline characteristics of the cohort (n = 422) were compared between continuers (n = 298, 70.6%) and discontinuers (n = 124, 29.4%) of rosuvastatin (Table 1). Factors significantly associated with discontinuation included statin initiation status (p = 0.0038), age (p < 0.0001), muscle symptoms (SAMS, p = 0.00572), and *ABCG2 rs2231142* (G>T) carriers (p = 0.00257). Discontinuers were more likely to be statin initiators (82.3% vs. 68.5%), younger (mean age: 47.53 vs. 53.64 years), and carriers of the *ABCG2 rs2231142 (*G>T) (30.6% vs. 17.4%). SAMS was reported in 111 patients (26.3% of the cohort), with a higher prevalence among discontinuers (35.5%) compared to continuers (22.5%). Among the entire cohort, 42.7% were on high-intensity rosuvastatin and 57.3% on moderate-intensity therapy, with no significant difference between groups (p = 0.648). Other characteristics, including sex, ethnicity, smoking status, thyroid disease, and *SLCO1B1* carrier status, showed no significant differences between the two groups. Based on the univariate analysis, the *SLCO1B1 rs4149056 (T>C)* variant did not show a statistically significant association with rosuvastatin discontinuation (*p* = 0.617). Therefore, this variable was excluded from subsequent multivariate analysis.

**TABLE 1:** Baseline characteristics and univariate association with rosuvastatin discontinuation.

Characteristic	Overall cohort (n = 422)	Continuers (n = 298)	Discontinuers (n = 124)	*P*-value continuers vs. discontinuers
Satin usage status				
Statin initiators	303 (71.8%)	204 (68.5%)	102 (82.3%)	**0.0038**
Pre-existing Statin User	119 (28.2%)	94 (31.5%)	22 (17.7%)	
Demographics				
Age, mean (SD)	51.84 (11.67)	53.64 (11.34)	47.53 (11.34)	**<0.0001**
Female, sex	161 (38.2%)	110 (36.9%)	51 (41.1%)	0.416
Arab Ethnicity	186 (44.1%)	135 (45.3%)	51 (41.1%)	0.431
Smokers	101 (23.9%)	74 (24.8%)	27 (21.8%)	0.502
Rosuvastatin dose				
High intensity	180 (42.7%)	125 (41.9%)	55 (44.4%)	0.648
Moderate intensity	242 (57.3%)	173 (58%)	69 (55.6%)	
Hypothyrodism	37 (8.87%)	27 (9%)	10 (8%)	0.741
SAMS	111 (26.3%)	67 (22.54%)	44 (35.5%)	**0.00572**
*ABCG2 rs2231142* (G>T*)* Carriers	87 (20.6%)	52 (17.4%)	38 (30.6%)	**0.00257**
*SLCO1B1 rs4149056* (T>C) Carriers	116 (27.5%)	84 (28.2%)	32 (25.8%)	0.617

Bold values indicate statistical significance.

### 3.2 Primary outcome: rosuvastatin discontinuation

**FIGURE 1:**
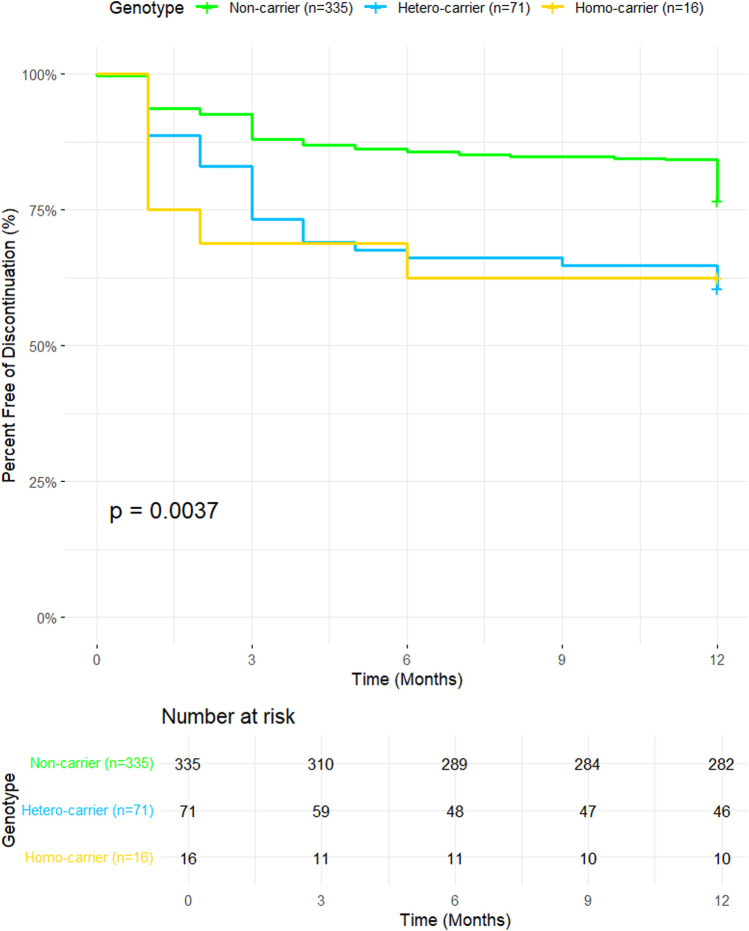
*Kaplan-Meier survival curves illustrating discontinuation-free survival over a 12-month period for participants with different ABCG2 genotypes.* The y-axis represents the percent free of discontinuation (%), and the x-axis represents time in months. Genotype groups include non-carriers (n = 335, green line), hetero-carriers (n = 71, blue line), and homo-carriers (n = 16, yellow line). The p-value (0.0037) indicates a statistically significant difference in discontinuation rates among the genotypic groups. The Number at Risk table below the plot shows the number of participants remaining at each time point for each group.

The Kaplan-Meier plot is shown in Figure 1. The orange line representing the *ABCG2 rs2231142* T/T group displays a steeper decline in the percentage of individuals free from discontinuation over time compared to the green line for the G/G group. This visual trend matches the HR of 1.75, indicating that participants with the G/T genotype have a 75% higher risk of treatment discontinuation compared to those with the G/G genotype (Figure 1).

The orange line representing the T/T group shows the steepest decline, which is consistent with the significant HR of 4.40. This indicates that individuals with the T/T genotype are more than four times as likely to discontinue treatment compared to those with the G/G genotype. The plot clearly shows that the T/T group has the lowest proportion of participants free from discontinuation over time, aligning well with the strong statistical significance observed (p < 0.001; Figure 1). This supports the significant differences observed between the genotypic groups.

### 3.3 Multivariate analysis of predictors of rosuvastatin discontinuation

Multivariate logistic regression models were used to estimate adjusted odds ratios (aORs), accounting for key predictors and potential confounders including ABCG2 genotype, SAMS, statin initiation status, and age. The presence of the *ABCG2 c.421C>A (rs2231142)* variant was significantly associated with an increased likelihood of discontinuation (adjusted OR = 1.861, 95% CI: 1.114–3.110, *P* = 0.018). Similarly, patients experiencing SAMS were at a higher risk of discontinuation (adjusted OR = 1.925, 95% CI: 1.189–3.118, *P* = 0.008), as shown in Table 2. Moreover, being a statin initiator was strongly associated with a higher risk of discontinuation (adjusted OR = 2.168, 95% CI: 1.275–3.687, *P* = 0.004). Conversely, age was a protective factor, with increasing age reducing the likelihood of discontinuation (adjusted OR = 0.958, 95% CI: 0.939–0.978, *P* < 0.001). (Table 2).

**TABLE 2:** Logistic regression analysis of factors associated with rosuvastatin discontinuation as a dependent factor.

Independent variables	P-value	Adjusted odds ratio (OR)	95% CI (Lower)	95% CI (Upper)
*ABCG2 c.421C>A (rs2231142)*	0.018	1.861	1.114	3.110
SAMS	**0.008**	1.925	1.189	3.118
Statin Initiators	0.004	2.168	1.275	3.687
Age	**<0.001**	0.958	0.939	0.978

**FIGURE 2:**
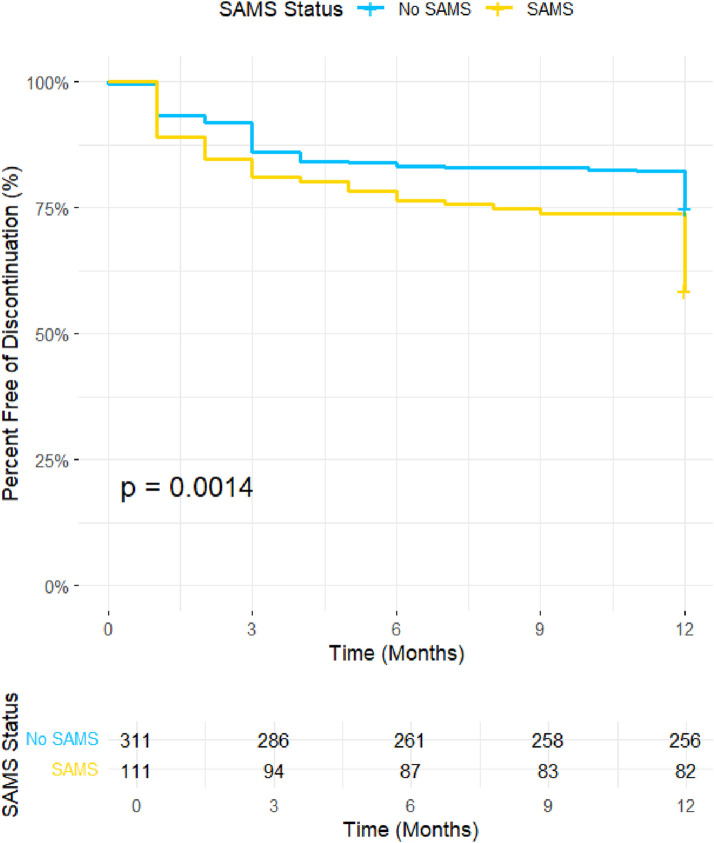
Kaplan Mier Curve illustrates the discontinuation-free survival probability over a 12-month period for participants with and without SAMS. The y-axis represents the percentage of participants free from discontinuation, and the x-axis represents time in months. SAMS; statin-induced muscle symptoms.

The impact of SAMS on treatment discontinuation was assessed using Kaplan-Meier survival analysis (Figure 2). Discontinuation-free survival was significantly lower in patients with SAMS compared to those without SAMS (p = 0.034). By the end of the 12-month follow-up period, approximately 72% of patients without SAMS remained on treatment, while only 66% of patients with SAMS continued therapy. These findings highlight the influence of SAMS on treatment persistence and suggest that the presence of muscle symptoms may substantially contribute to the early discontinuation of rosuvastatin therapy.

### 3.4 Variability in LDL-C response by discontinuation status

**FIGURE 3:**
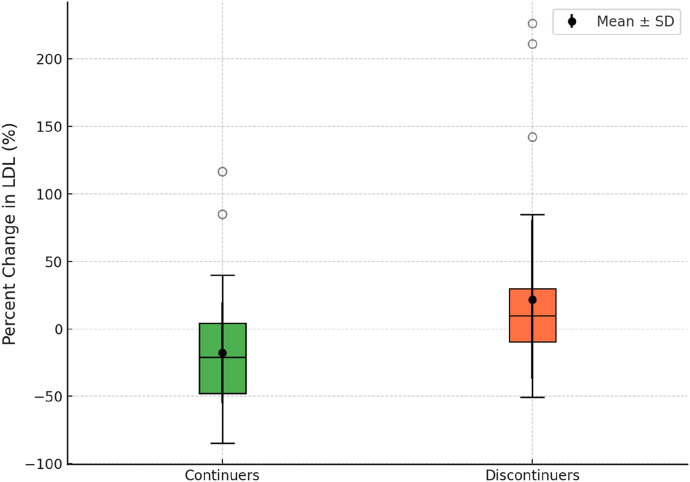
Box plot illustrating the percent change in LDL between rosuvastatin continuers and discontinuers.

Moreover, we assessed the mean percent change in LDL cholesterol levels between continuers and discontinuers. Continuers (n = 61) demonstrated a mean percent LDL reduction of -17.86% (SD: 37.51, range: −85–116.6), while discontinuers (n = 39) exhibited a mean percent LDL increase of 21.89% (SD: 58.96, range: −51–226). The difference between the two groups was statistically significant (p < 0.001). These findings are visually represented in Figure 3, which illustrates the distribution of percent change in LDL cholesterol levels between the two groups.

## 4 Discussion

This study highlights the *ABCG2 rs2231142* *G>T* genetic variant as a significant determinant of treatment continuation among rosuvastatin users. It also emphasizes the impact of SAMS on treatment continuation and the potential effect of discontinuation on LDL levels.

The *ABCG2 rs2231142* *G>T* genetic variant is linked to higher plasma levels of rosuvastatin, which increases the likelihood of adverse effects, specifically muscle-related issues (Song et al., 2022). This variant leads to reduced activity of the *ABCG2* transporter, hindering the elimination of rosuvastatin from hepatocytes into bile and resulting in greater systemic drug exposure (Keskitalo et al., 2009). Meta-analysis of Song et al. (2022) included 423 participants across eight studies that demonstrated that the *ABCG2 c.421C>A* variant significantly increases rosuvastatin plasma levels in T allele carriers (Song et al., 2022).

Other research indicates that individuals with the *ABCG2 c.421C>A* variant face a heightened risk of rosuvastatin-related side effects, potentially leading to an increased rate of therapy discontinuation and non-adherence (Bradley et al., 2019; Lehtisalo et al., 2023). Our Kaplan-Meier survival analysis demonstrated that participants with the TT genotype had the steepest decline in treatment persistence (HR: 4.40; 95% CI: 2.07–9.33; p = 0.0001), followed by those with the GT genotype (HR: 1.75; 95% CI: 0.99–3.09; p = 0.0547). These findings indicate that the TT genotype is associated with a more than fourfold increased risk of treatment discontinuation compared to the GG genotype. At the same time, GT carriers exhibited a 75% higher risk. Patients with the TT genotype, representing the homozygous minor allele, were only 16 out of 422 participants; these findings may not be fully generalizable to non-UAE populations due to differences in allele frequencies. Our findings highlight demographic factors associated with treatment persistence. Furthermore, statin discontinuers were significantly younger than continuers (mean age 47.53 vs. 53.64 years; p < 0.0001), consistent with studies indicating that younger patients often perceive lower cardiovascular risk, leading to reduced adherence (Vinogradova et al., 2016). This finding aligns with previous studies showing that younger patients are often less adherent to chronic therapies (Sütlü, 2023; Cohen and Mykyta, 2024), possibly due to lower perceived cardiovascular risk (Pettersen and Candelaria, 2024). A statistically significant difference was observed in the proportion of statin initiators between continuers and discontinuers (p = 0.0038), with discontinuers more likely to be new users. This trend suggests that patients initiating statin therapy may face greater challenges with tolerability (Patel et al., 2016). Other variables, such as rosuvastatin dose intensity or comorbidities like thyroid disease or smoking status, did not show significant associations with discontinuation. This suggests that genetic and demographic factors may have a more pronounced impact on continuation than clinical variables alone. The systematic review of 34,196 participants demonstrated the superior LDL-lowering efficacy of rosuvastatin over atorvastatin, reinforcing its value as a high-intensity statin (Jaam et al., 2023). However, its effectiveness in real-world settings may be compromised by treatment discontinuation due to adverse effects.

Our previous observational study evaluated the association between muscle pain, elevated liver enzymes, and several factors, including genetic testing results. Building on this work, the current study shifts focus to test the association between discontinuation, *ABCG2* minor allele carriers, and experiencing muscle pain. Building on existing evidence regarding atorvastatin, particularly the role of the SLCO1B1*5 allele in increasing the risk of statin-associated musculoskeletal symptoms (SAMS) and subsequent treatment discontinuation (Voora et al., 2022), this study addressed this gap by examining the association of genetic variants, including *SLCO1B1 rs4149056* and *ABCG2 rs2231142*, with rosuvastatin-related adverse effects and discontinuation rates over 12-month follow-up period. The analysis of statin-associated musculoskeletal symptoms (SAMS) reveals significantly lower rates of treatment continuation in patients who developed SAMS compared to those who did not (p = 0.034). This demonstrates that clinical adverse effects like SAMS are a critical determinant of the continuation of rosuvastatin therapy (Thompson et al., 2021). To mitigate SAMS and enhance adherence, clinicians should employ patient-centered strategies, including managing expectations, addressing nocebo effects, emphasizing the benefits and safety of statins, and optimizing lifestyle interventions to potentially lower the required statin dose, change statin type, with evidence showing 60%–80% of patients with SAMS can eventually tolerate one statin regimen. Still, it has to be the right one (Warden et al., 2023). By proactively addressing both genetic and clinical risk factors, clinicians can mitigate the risks of SAMS, reduce discontinuation rates, and optimize therapeutic outcomes. These results reinforce the critical importance of personalized approaches in managing rosuvastatin therapy, particularly in diverse populations with variable allele frequencies. The demographic and clinical characteristics presented in the table provide valuable context to the observed differences between continuers and discontinuers of rosuvastatin therapy. consistent with the Kaplan-Meier findings demonstrating higher discontinuation rates in the first 3 months among those with adverse effects, such as SAMS. These findings underscore the significance of considering *ABCG2* genotypes in personalizing rosuvastatin therapy to optimize efficacy and minimize adverse effects. Furthermore, a meta-analysis of 34,150 participants demonstrated that the A allele is linked to significant changes in the lipid profile, including lower HDL-C levels and higher LDL-C and total cholesterol levels, contributing to an increased risk of dyslipidaemia, particularly in Asian populations (Liu et al., 2024). While the A allele enhances the lipid-lowering efficiency of rosuvastatin due to reduced efflux of the drug by the ABCG2 transporter, the resulting increased systemic exposure raises the likelihood of side effects, leading to a higher risk of treatment discontinuation. These findings underscore the importance of considering the *ABCG2 c.421C>A* variant in personalized lipid management to optimize both efficacy and tolerability. *The ABCG2 c.421C>A* variant was significantly more prevalent among discontinuers (30.6% vs. 17.4%, p = 0.0026). Interestingly, no significant differences were found in other variables, such as rosuvastatin dose intensity or the prevalence of comorbidities like thyroid disease or smoking status, indicating that genetic and demographic factors may play a more central role in driving discontinuation rates than clinical variables. These findings collectively support the conclusion that genetic predisposition, particularly the *ABCG2 c.421C>A* variant, is a critical determinant of treatment persistence, potentially through its role exacerbating adverse effects like SAMS. Integrating these insights into clinical practice can improve adherence and optimize therapeutic outcomes, particularly in diverse populations with variable allele frequencies. The logistic regression results indicate that discontinuation of treatment significantly increases the odds of LDL decrease by more than 10% (Odds Ratio = 4.805, *p* < 0.001), suggesting a strong association between discontinuation and the outcome. In contrast, Rosuvastatin intensity (Odds Ratio = 0.552, *p* = 0.183) and muscle aches (Odds Ratio = 0.795, *p* = 0.642) did not show significant effects on the outcome, indicating their weaker or non-significant relationships with LDL decrease.

The observed 21.89% increase in LDL-C following drug discontinuation is clinically significant, as elevated LDL-C is a well-established risk factor for cardiovascular disease (CVD). Large-scale meta-analyses, including the Cholesterol Treatment Trialists’ (CTT) Collaboration, have demonstrated that each 1 mmol/L (∼38.67 mg/dL) increase in LDL-C is associated with a 20%–25% higher risk of major cardiovascular events (Cholesterol Treatment Trialists’ Collaboration, 2010). Given this relationship, a 21.89% increase in LDL-C from a baseline of 100 mg/dL (∼2.59 mmol/L) translates to an increase of ∼21.7 mg/dL (∼0.56 mmol/L), which corresponds to an estimated 11%–14% increase in CVD risk. This effect may be more pronounced in high-risk populations, including individuals with diabetes, hypertension, or pre-existing atherosclerosis. Discontinuation of lipid-lowering therapy can therefore accelerate atherosclerotic plaque progression, predisposing patients to myocardial infarction and stroke (Ference et al., 2017). These findings emphasize the importance of maintaining lipid-lowering therapy e and, in cases where statins are discontinued due to adverse effects, considering alternative agents such as ezetimibe or PCSK9 inhibitors to mitigate the heightened cardiovascular risk (Bosworth et al., 2018).

Clinically, this variant has been linked to fluctuations in treatment outcomes. For instance, a study involving 305 Chinese patients with hypercholesterolemia found that carriers of the 421A allele experienced a greater reduction in low-density lipoprotein cholesterol (LDL-C) levels when treated with rosuvastatin, indicating enhanced drug efficacy (Wan et al., 2015). Although the study provides valuable insights, it has several limitations, including the inability to consistently determine the precise date of discontinuation, as many patients could not recall the exact timing. Future studies with larger sample sizes and a higher proportion of individuals homozygous for the T allele are needed to confirm and expand upon these findings, given that only 16 such individuals were included in our current cohort. Increasing the number of homozygous T allele carriers would enhance the statistical power to detect genotype-specific effects. Additionally, complete LDL data were available for only 100 patients, which may limit the generalizability of findings. Further, potential confounding factors such as lifestyle modifications, adherence monitoring methods, and variations in clinical practices were not assessed, which may have influenced treatment persistence and discontinuation rates. While the *ABCG2 c.421C>A* variant may enhance rosuvastatin efficacy, it also poses challenges due to increased discontinuation rates. Personalized treatment strategies, including genetic screening and patient education on the importance of adherence, are essential to optimize outcomes in hypercholesterolemia management.

## Data Availability

The data presented in this study are not publicly available due to participant privacy concerns and data sharing restrictions set by the institutional ethics board. De-identified data may be made available upon reasonable request to the corresponding author, subject to ethical approval.
